# What is the process by which a decision to administer electroconvulsive therapy (ECT) or not is made? A grounded theory informed study of the multi-disciplinary professionals involved

**DOI:** 10.1007/s00127-018-1541-y

**Published:** 2018-06-08

**Authors:** Anna Duxbury, Ian Smith, Bethan Mair-Edwards, Gerry Bennison, Kerry Irving, Suzanne Hodge, Ian Anderson, Stephen Weatherhead

**Affiliations:** 1 Department of Clinical Psychology, Lancaster University, Lancaster, UK; 2 Liverpool, UK; 3 Neuroscience and Psychiatry Unit, University of Manchester, Manchester Academic Health Science Centre, Manchester, UK; 4 Department of Clinical Psychology, University of Liverpool, Room 2.12, Whelan Building, Brownlow Hill, Liverpool, L69 3GB UK

**Keywords:** Grounded theory, Staff, Electroconvulsive therapy, ECT, Decision making

## Abstract

**Purpose:**

To develop a grounded theory-informed model explaining the decision-making process professionals in multi-disciplinary teams go through in deciding whether to administer electroconvulsive therapy (ECT) or not.

**Methods:**

A grounded theory informed methodology was used to analyse the data offered by ten participants who had all been involved in the process of deciding if someone has ECT or not.

**Results:**

The core categories, described as ‘layers’ in this research, ‘personal and professional identity’; *‘*subjective vs objective’; *‘*Guidelines or Clinical Instinct?’; ‘Someone has to take Responsibility’ and ‘the decision in action’, were constructed from the data.

**Conclusions:**

The study describes a useful insight into the layers of the decision-making process that could be further considered in clinical settings. The model highlights the decision to give ECT that has many different layers including professional identity, how a person understands the evidence base, past experiences, and the amount of power they have in the process. The consultant psychiatrist and the patient were seen as holding most power in the process depending on whether the Mental Capacity Act (2005) or Mental Health Act (2007) was being followed. Patients were seen to experience a very different decision-making process dependant on the personal views of the professionals in relation to ECT.

## Introduction

Electroconvulsive therapy (ECT) involves passing an electric current briefly through the brain which then induces generalised seizure activity [1]. The Royal College of Psychiatrists’ (RCPsych) ECT Handbook states “75 years after its introduction, ECT remains the most effective treatment for severe depressive disorder” [2, p. 1] and in the United Kingdom (UK), many policymakers and psychiatrists regard ECT as an effective intervention [3]. The literature surrounding ECT, however, is often polarised [4–6]. For example, Read and Bentall’s [4] systematic review concluded ECT causes “significant increased risk of death” and “the cost-benefit analysis for ECT is so poor that its use cannot be scientifically justified” [4, p. 333]. Whereas, the UK ECT group [5] meta-analysis concluded ECT is significantly more effective than “simulated ECT” and pharmacotherapy with a large effect size.

The National Institute for Health and Care Excellence (NICE) recommends ECT be considered for moderate to severe depression, not responding to other treatment [7, p. 3]. The ECT Minimum Dataset Activity Data Report 2015 [8] and the Scottish ECT Accreditation Network (SEAN) Annual Report 2015 [9], however, show although depression is the most common reason for referral, ECT is used to treat other forms of psychological distress, including experiences categorised as: “adjustment disorder, borderline personality disorder, emotionally unstable personality disorder and peri-natal depression”.

### ECT policy and legislation

The SEAN reported (2016) 60% of patients receiving ECT were informal and capacitous [9]. The ECT Minimum Dataset Activity Data Report (2015) reported 51% of patients were informal and had capacity to consent to treatment at the start of the course [8]. The report also stated 81.6% of people who received ECT whilst detained under the Mental Health Act [MHA] (2007) in 2014–2015 did not consent, due to being deemed unable to give valid consent, at the start of the treatment.

According to the MHA (2007), if a person is capable of understanding the nature, purpose and likely effects of the treatment, they have capacity to make a decision and ECT cannot be given without their consent. ECT can only be given to a person who is deemed to not have capacity when an independent, specially approved psychiatrist has authorised it. If a person deemed as having capacity refuses treatment, the only circumstance in which ECT can still be administered is under section 62 (1A & 1B) of the MHA (2007) which is an emergency treatment usually because there is a fear the person will die if they do not have the treatment.

The Mental Capacity Act (MCA) (2005) runs alongside the MHA in England and Wales, providing a statutory framework for decision making with adults who may lack the capacity to make specific decisions for themselves. The core purpose of the MCA is to empower individuals to make their own decisions wherever possible, as well as protecting vulnerable individuals who lack decision-making capacity. The MCA (2005) specifically includes ECT, to ensure there is a safeguard in place for people who lack capacity to consent to ECT, but not detained under the MHA.

Given the high proportion of cases in which ECT is administered under the MHA without consent, it is crucial we understand how decisions are being made. There has been little research exploring staff views on the use of ECT. One study found there were significant differences in attitudes towards ECT between those in different job roles [10]. The study highlighted a need for awareness of differences of opinion within multi-disciplinary teams (MDT) towards the treatment, stating teams should be aware there might be strong differences of opinion amongst members. It is important for the development of clinical care, to understand how the decision-making process is taking place so mental health professionals can ensure as many people as possible are consenting to the treatment. The current study will look to address the following research question: what is the current decision-making process, with regards to ECT administration, from the different professional perspectives involved?

## Method

### Design

A grounded theory informed design was used to enable in-depth exploration of participants’ experiences of ECT decision-making processes. Grounded theory was first postulated by Glaser and Strauss [11]. Since then three main versions of grounded theory have emerged, Charmaz [12] describes them as objectivist [11], post-positivist [13] and constructivist [13]. The current study adopted Charmaz’s [14] approach. This assumes theories are not discovered, but rather constructed through the research process, and the model created will map perceptions of processes in its theories, rather than the underlying realities. While objective versions of grounded theory state the researcher needs to ‘ignore’ previous data, with the positivist view that reality can be captured objectively as it is, Charmaz, and others [e.g. 15], believe the researcher should acknowledge their position and prior knowledge in relation to the topic being studies [14].

### Ethics

UK NHS ethical approval was obtained and local NHS Research and Development guidelines were adhered to.

### Sampling and participants

Participants were recruited from two large NHS Trusts, across multiple sites. To gain access to a wider pool of participants, three amendments were sought to the initial research governance approvals to recruit participants via social media platforms. A total of ten participants were recruited (Table 1).

**Table 1:** Demographic information of participants

Name	Age	Ethnicity	Job role	Gender
Participant (P) 1	35	–	Clinical Psychologist (older adults)	Female
P2	54	White British	Specialist ECT nurse practitioner	Female
P3	50	White British	Ward Manager (acute mental health ward)	Male
P4	–	–	Consultant Psychiatrist (acute mental health ward)	Male
P5	24	White British	Deputy Ward Manager (acute mental health ward)	Female
P6	42	Indian	Locum Consultant Psychiatrist (acute mental health ward)	Male
P7	51	White British	Lead ECT nurse	Male
P8	35	White British	Support time recovery worker (community mental health)	Male
P9	29	White British	Clinical Psychologist (acute mental health ward)	Female
P10	–	White British	Advanced Practitioner (acute mental health ward)	Male

It was also intended to ask about reason for referral, mental health act status at time of ECT and number of ECT treatments; however, as these were not applicable for any of the recruited participants they have not been added to the table

Where data are missing this is either because the data were not provided by the participant or as a safeguard to protect anonymity

### Data collection and analysis

All interviews were conducted and analysed by the first author. Each participant was interviewed once, using a flexible topic guide. The topic guide developed as data collection and analysis progressed in line with Charmaz [14]. Interviews lasted between 28 min (final interview) and 80 min.

Initial interviews were undertaken with four participants; clinical psychologist, consultant psychiatrist, ECT lead-nurse and ward manager. All interviews in the study were conducted face to face. Memos and reflective notes were written after each interview to capture ideas the researcher had about initial codes emerging from the data. The initial four transcripts were then line-by-line coded [14]. Memos were again written at this stage to ensure the researcher was analysing the ideas early on in the research process and to increase the level of abstraction in the ideas [14]. Codes were amalgamated to form focused codes and then further amalgamated, utilising the researcher’s memos, to form conceptual codes. The findings were then used to adapt the topic guide [14]. The process highlighted participant demographics to focus on, in line with theoretical sampling [14], to develop the emerging conceptual codes further.

A further six participants were recruited and analysis repeated in constant comparison with the initial codes and conceptual categories. If data emerging in the interviews did not fit with existing initial codes, new codes were developed. After this second round of data collection and analysis, data sufficiency was considered to have been achieved [16]. That is, the conceptual categories developed did not require revision or alteration in respect of new data. Final, conceptual categories were developed to form a grounded theory informed model of the decision-making process.

### Reflexivity

As the version of grounded theory used encouraged the researcher to acknowledge their position and prior knowledge in relation to the topic being studies [11], the literature discussed in the introduction was reflected on throughout the process. Reflections were made on the first authors’ perspective of, and experience of conducting the ECT research, on an Internet blog site. As this research was conducted as part of a Doctorate in Clinical Psychology, reflections were made and discussed in monthly research supervisions and within a separate research involvement group. Reflecting on these experiences enabled acknowledgement of assumptions and biases within the research process. An example of a bias discussed was how the author would manage, and resist, a potential bias to conform to the common ‘anti ECT’ narrative in clinical psychology.

### Findings

**Fig. 1:**
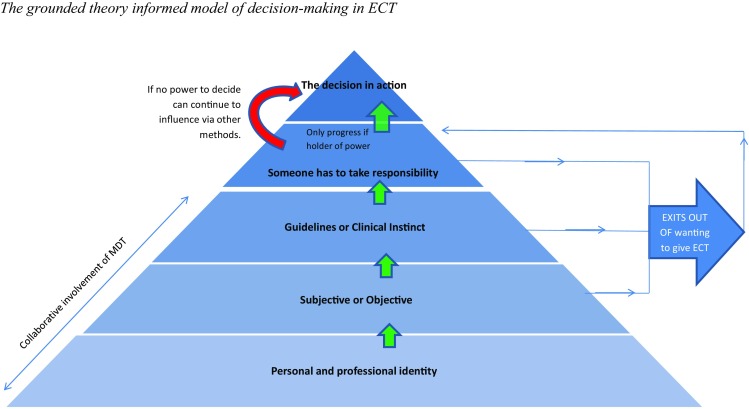
The grounded theory informed model of decision making in ECT

Findings should be read alongside the model in Fig. 1. The model shows the stages involved in making a decision to give ECT or not, as represented by a pyramid with the decision in action at the pinnacle. Each layer of the pyramid shapes the layer above it. If a person thinks ECT should be given then they progress to the next layer. At each layer are exits from the decision-making model which would result in a decision not to give ECT. The first layer, *the personal and professional identity*, highlights the boundaries of the model.

### Subjective or objective

Most participants talked about the treatment improving mood. Harmfulness was described in terms of side effects to ECT (nausea, vomiting, headaches), contraindications (such as cardiac problems) that may result in serious harm or death for the patient if they were to be given ECT, and cognitive impairments as a result of the ECT.

All participants had witnessed some form of “dramatic” improvement in individual patients and this undoubtedly influenced their subsequent decision making. Any difficulties they perceived as being potentially caused by giving someone ECT, particularly against their will, were often seen as being mitigated by these improvements.

Even though it is horrible at first and you do have to put hands on and take someone round there you can justify it in your head because you think I know it will get better for that person and so that’s how I have rationalised it before [P5, Deputy Ward Manager (DWM)].

However, participants said some professionals would never give ECT: “we know that there are certain consultants within the Trust who don’t like ECT … some consultants just don’t prescribe ECT at all” (P7, Lead ECT nurse). Some felt this was harmful to patients as they were being deprived of a treatment that could potentially help before they had even had chance to consider it, whereas others thought this decision making was acting in the best interest of patients as it was withholding a harmful treatment.

### Guidelines or clinical instinct

All but one of the participants felt ECT should only be given after other options had been tried first: “I mean I don’t think we should jump straight to ECT” (P6, Consultant Psychiatrist). The majority of the participants spent most of the interview discussing ECT in regards to life-threatening situations or in regards to severely distressed people who were “stuck” in their distress. It was difficult to prompt conversations about what happened before people reached this point:

Not to sound like a broken record but it’s the lifesaving thing. It is the only thing that I think we can do to genuinely save a life. For most other things you can contain and eventually you will get there but for that group of people you have to do something [P10, Advanced Nurse Practitioner (ANP)].

When asked why ECT was not considered in the first instance the participants cited examples of side effects such as memory loss, confusion, headaches and nausea. There was also an idea of ECT being seen as “invasive”, “intrusive”, or even “barbaric”. This intrusive element left participants feeling uncomfortable yet it was very difficult for the participants to elaborate on this feeling. This “uncomfortable feeling” (P10, ANP) could be alleviated if there was trust in the effectiveness of the treatment “well it’s usually the same presentation, i.e. if they are, especially if they have had it before, then I know it is going to work that they’ll get better.” (P7, Lead ECT nurse).

When participants described people who they felt should be considered for ECT they described them as “catatonic” or “severely depressed”. Participants spoke mainly in diagnostic language; however, they explained further what those diagnostic presentations would look like in practice: “not eating”, “not drinking” and “just existing” were common terms used to describe people.

Some of the participants made reference to guidelines [NICE & Electroconvulsive Therapy Accreditation Service (ECTAS)] that supported their decision of who was appropriate to receive ECT. However, in the main, people stated the decision came from clinical observation and prior experiences.

We don’t go off that [guidelines] I think as nurses what we’d be looking for is observational signs. So if we see somebody come in and you know and we are a ward that does persevere and we try and get them motivated, try and get them to do things and if we just, if those signs and those alarms bells are going that’s when we would bring it to the table (P5, DWM).

Although many participants focused on physical harm some also considered psychological harm.

I knew that that was a particular difficult topic for him [having ECT] because his dad had ECT around the same age and so that was part of the formulation. There was a lot of that feeding in to like a feeling of a self-fulfilling prophecy really (P9, ClinPsy).

The predicted psychological harm ECT could cause was something that could cause a staff member to rule out giving someone ECT. However, if life was in danger then ECT all staff felt it should be used regardless of the potential psychological harm caused by the treatment. In this sense, some participants appeared to see psychological distress as something different from the “illness” the ECT was treating or a sense that more psychological harm would be caused/maintained by not treating the depression. Effectiveness for these participants was ECT ‘treating’ the person so they started eating or drinking again. Effectiveness was viewed as boosting a person’s motivation to engage with life again. Psychological harm caused by the treatment (e.g. as a result of being restrained) was something that needed to be addressed, but at a later stage. It was also something not addressed enough according to some participants:

I think at the time it probably is the right treatment to do from a life-saving point of view but then we have also got to consider that it is quite, I’d say, parental. It is going back to the old days of strapping them to the bed and giving them a treatment and all the ensuing memories that could dredge up. I don’t necessarily think that we address that (P10, ANP).

Feelings associated with nursing or caring for a person in so much distress was also distressing and appeared to be a factor that influenced how people made their decision: “well it is awful. It’s awful… this job it will emotionally pull you. We are only human.” (P5, DWM).

### Someone has to take responsibility

Participants weighed up their own opinions on whether ECT should be given or not. Some participants exited the model at this point, concluding ECT was not the best option for the patient. The participants, however, held different amounts of power and, therefore, confidence their own decision would be followed. There was certainty in all of the participants’ accounts that if a patient had capacity then it was for the patient to make the decision to have ECT or not. “If they are informal [and don’t want ECT], they don’t get it. We don’t treat. It’s as simple as that” (P2, Specialist ECT nurse). This view of capacity was the same even if the person was detained under the MHA (2007); “then they don’t have it. If they are under a section and they have capacity and they are not consenting then they will not be having it!” (P7, Lead ECT Nurse).

If the patient did not have capacity to decide, participants indicated a MDT decision should be made about whether the patient was to receive ECT or not, but suggested decisions were more individually led: “yeah it has to be a team decision. Well it has to be a team discussion, I’m not saying it is a team decision but it has to be team discussion” (P5, DWM). The team decision was said to be dependent on the consultant psychiatrist whom all ten participants described as having ultimate power in the process:

I think it comes down to the personality of the consultant and whether that is someone who values the skill, experience, opinion of their team because my experience of working on the wards is that some consultants really appreciate and absorb that and other don’t (P9, ClinPsy).

Under the MHA (2007) the consultant psychiatrist is named as the Responsible Clinician, which can be perceived as forcing the consultant to rely on their own decision-making process rather than take account of the opinions of others.

I think the problem that we have got is that our consultant had a few like serious incidents where people have gone and killed themselves so his anxieties is, it’s awful when you are standing in front of a coroner…but the way that he sees it is that [they] have to do everything that [they] can in order so that if someone does then they have tried everything (P5, DWM).

All the consultants in the study stated they were ultimately responsible for the decision. However, they also suggested the decision to give ECT was made in collaboration with their MDT and felt there were never disagreements around the decision: “I don’t think we have ever had a case where I wanted ECT and the nursing staff hasn’t.” (P6, Consultant Psychiatrist). There was also a sense other members of the team looked to the consultants for answers.

### The decision in action

Once it had been decided who held the power to make the decision, the decision was put into action.

*Advocating* The participants talked about times when the patient did not have capacity or when the patient complied with a consultant’s decision even if they did not agree with it.

I want to do the best job that I can … And if that means standing up to a consultant because we have been advocates for patients in the past and it hasn’t all been smooth (P2, Specialist ECT Nurse).

Participants also talked about how some people, particularly older patients, would never challenge the views of the psychiatrists and even if they did have capacity they would often go along with what the consultant was saying. In these situations, the participants felt they had to advocate on behalf of the patient. This was driven by a need to either promote the patient’s autonomy, or promote the participant’s opinion over the decision made because they felt it was in the patient’s best interests to make their own decisions.

*Reassure/persuade* A patient may have held the power to say no to the treatment; however sometimes the participants still believed the treatment was the best option for the patient. In these situations, many of the participants saw a main part of their role being to reassure patient ECT was a good treatment choice: including showing people around the ECT suite “we also facilitate patients coming down to the unit before they are treated so they can have a look around if necessary” (P2, Specialist ECT Nurse); alleviating stigma “well first off it is trying to alleviate the stigma of ECT: the myth about it being a painful process which it is not” (P4, Consultant Psychiatrist), and talking people carefully through the process:

Tell them [the patient] what the treatment entails, probably what will happen, going through the process so they know. We try to give them as much reassurance as possible. That also is, it’s like, a face to face so they will have, so that they will know us when they are here (P2, Specialist ECT Nurse).

The majority of the professionals who engaged in these types of actions labelled them specifically as reassurance and maintained it was ultimately the patient who made the final decision. The participants reasoned this reassurance was necessary because they believed the patients started the process with a very negatively biased image of ECT:

I think there is a lot of stigma around it and I think especially with films and what they have seen. It’s completely different and they can go and have a look around the ECT suite if they want to, they can have all that information so they can see it’s not this barbaric treatment (P5, DWM).

Other participants saw reassurance as persuasion; “…they would discuss the risks, wouldn’t they, but they were basically trying to persuade him to… from what he says it was kind of like pressure, and pressure really to have it” (P9, ClinPsy). One participant specifically said he felt his role was to convince people to have ECT if he felt the treatment was in the patient’s best interests “but I think it is my job to convince even those who might not want it if I think in my view they might benefit from ECT.” (P6, Consultant Psychiatrist). This seemed to come from a paternalistic position of knowing what was in the best interest for the patient to promote wellbeing, despite the patient having the capacity to make their own decision. Participants who held such views tended to see ECT as a “solution” that would eliminate the suffering of the patient. Others who felt distress should be “sat with” and would not just be solved, leaned away from feeling the need to persuade a person to have the treatment.

*Enabling autonomy* The final option for action was supporting the patient to make an authentic, informed, autonomous decision. This was when the patient held the power to make their own decision. This action was difficult to tease apart from reassurance, as many of the participants believed they were also supporting choice when they were offering reassurance to a client. All the professionals said it was important to them that the patient is allowed to make an informed choice. This was most notably important for the participants in the study who had experienced times when the patient was not as involved in their care:

I started off my career with a fairly negative view of it [ECT] really. I tended to think, and maybe I still do, that it was given to elderly people and by default those people at the time were subservient to doctors; they would do what the doctor said and that made me a little uncomfortable about that. There also seemed to me, people who were disenfranchised in some ways got ECT sooner than other people so people without advocacy, people without family, they got ECT sooner than other people so that made me uncomfortable (P10, ANP).

**Fig. 2:**
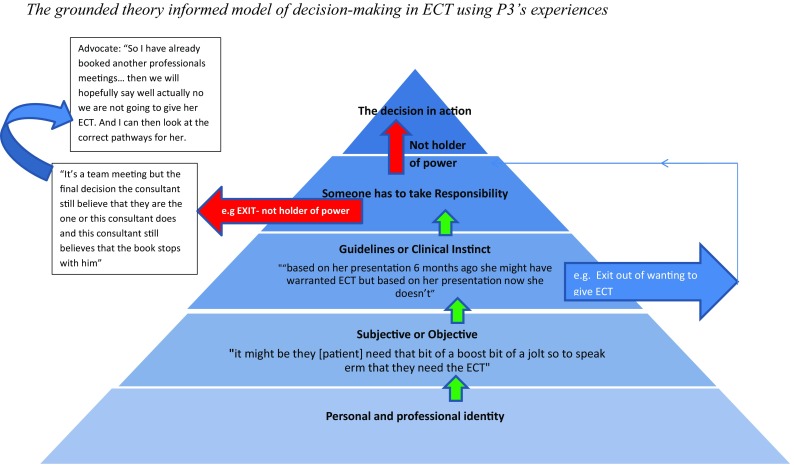
The grounded theory informed model of decision making in ECT using P3’s experiences

Ways to ensure patients had more ‘say’ in their treatment included offering advocacy and having more family carer involvement. Psychological support was rarely offered to the patient. Having a psychological formulation that supported a patient to understand why they were “stuck” in their distress was discussed as being important to help a patient make an informed, autonomous decision about whether they wanted ECT (Fig. 2).

## Discussion

The findings have begun to explore the individual process of deciding whether to give ECT or not. Individuals go through stages of decision making represented by the layers of the grounded theory model (Fig. 1). Each layer is advanced when a person decides, by considering numerous factors, ECT may be the best option for the patient. Each layer can be advanced until the “somebody has to take responsibility” layer: which is only progressed by the consultant or the patient as legally stated by the MHA (2007) and the MCA (2005).

Consultants have the current UK’s working version of the Hippocratic Oath [17] as their guiding principle in ECT decisions. Some doctors utilise the evidence base and previous experience to offer ECT in keeping with the Hippocratic Oath, whereas others, who utilise the evidence base and previous experience, conclude ECT is harmful and follow the Hippocratic Oath by not offering ECT. Indeed, the General Medical Council’s [14] guidance states doctors “must use your judgement in applying the principles to the various situations you will face as a doctor”.

NICE holds shared decision making as a core part of the development and implementation of guidelines, as defined by three components:

- care or treatment options are fully explored, along with their risks and benefits,
- different choices available to the patient are discussed,
- a decision is reached together with a health and social care professional [18].

When applying this to ECT, there are two significant challenges. First, at the point of undergoing ECT, many people lack the capacity to engage in a shared decision-making process [8]. Second, there is limited time given to developing a formulation-driven approach to mental health treatment [19]. Consequently, whilst NICE [20] advocate for shared decision making in mental health there is an observable difference in how clinical decisions are made in community versus acute settings [21]. Regardless of setting, ethical principles of beneficence and non-maleficence, autonomy and justice may still be applied. The question is one of how they are applied to move beyond “the assumption the clinician is the only competent decision maker, who will make decisions for rather than with the patient” [22].

A United Nations General Assembly Special Rapporteur on human rights and the mental health system noted “power imbalances reinforce paternalism and even patriarchal approaches, which dominate the relationship between psychiatric professionals and users of mental health services. That asymmetry disempowers users and undermines their right to make decisions about their health, creating an environment where human rights violations can and do occur” [23].

Within the present research, participants often decided to give ECT because it would make the person “better” and, therefore, lead them to “recover” from their “episode of illness”. Recovery is a “poly-variant” concept with understandings offered from biomedical, social and service user-led perspectives [24]. The different professional perspectives on ECT [10], as discussed in the introduction, may also be applied to perspectives on recovery. ECT is, however, a predominantly biomedical approach. Indeed, some participants in the study said the decision to give ECT was a wholly medical decision that did not need to include non-medics. Recovery in a biomedical sense commonly refers to response and remission (indicating degrees of relief of symptoms), which is narrower than the concept of recovery. When used they imply the regaining of function, which is different to poly-variant concepts of recovery as a process of living with, and working on, the problem. Indeed, participants suggested this ‘biological recovery’ comprised an improvement in mood from the excitation of the nervous system and a reduction in the risk of dying from not eating or drinking. ECT, therefore, although could be argued to offer response and remission it perhaps cannot offer the prospect of recovery. This suggests an argument if ECT is going to be used then an understanding and interventions from other approaches (e.g. social and psychological approaches) need to be taken into consideration alongside ECT too.

Participants stated the power to decide if they wanted ECT was always with the patients as long as they had the capacity. This is consistent with previous studies referenced in the introduction, which place human rights at the fore of decision making in ECT [1]. However, anecdotal evidence suggests Advance Directives are not always used meaning a person’s wishes, when in a capacitous mind, are not always known. Even if they are, from a legislative perspective it is always possible a person’s physical deterioration is such that ECT will be prescribed as a life-saving intervention. This responsibility for keeping someone alive and the consequences of not doing, may lead to a risk-averse approach.

## Future research

Future research should look to further interrogate the decision-making model presented here and extend the model with service users and carers. Building on the current model would show if patients and carers agreed with the current model suggestion that advocating, reassuring/persuading and informing occur, and how they influence a patient’s decision making.

Given this was the first study looking to explore the decision-making process involved in ECT, it aimed to cover all aspects of the decision-making process. The data highlight a complex decision-making process which appears to have related but distinct processes for if a patient has capacity and if they are deemed not to, or if the patient is detained under the MHA or as an informal patient. The processes for each of those categories should be further explored.

Research to date, as explored in the introduction, has tended to draw a distinction between symptom-based and experience-based empirical research. The present study synthesises them to produce a grounded theory of how legislation is applied in practice and how people utilise all forms of ‘evidence’ to reach and reflect on treatment decisions. Given Electroconvulsive Therapy is a controversial treatment with polarised opinions from professionals and patients, it is important there is consistency and transparency in the decision-making process. Furthermore, it is important we continue to ensure as many people as possible are able to consent to the treatment. This paper has attempted to begin the conversation on how we best achieve that.

## References

[1] Barnes R (2015). Information about ECT (Electro-convulsive therapy) [leaflet].

[2] Anderson IM, Fergusson GM, Waite J, Easton (2013). Mechanism of action of ECT. A the ECT handbook.

[3] Singhal A (2011). Electroconvulsive therapy and its place in the management of depression. Progr Neurol Psychiatry.

[4] Read J, Bentall R (2010). The effectiveness of electroconvulsive therapy: a literature review. Epidemiol E Psichiatria Sociale.

[5] UK ECT Group (2003). Efficacy and safety of electroconvulsive therapy in depressive disorders: a systematic review and meta-analysis. Lancet.

[6] Sienaert P (2011). What we have learned about electroconvulsive therapy and its relevance for the practising psychiatrist. Can J Psychiatry.

[7] National Institute of Clinical Excellence (NICE) (2009). Technology appraisal guidance 59: guidance on the use of electroconvulsive therapy.

[8] Royal College of Psychiatrist (2015) ECT minimum dataset activity data report—England & Wales. https://www.rcpsych.ac.uk/pdf/ECT%20Minimum%20Dataset%20Report%202014-15.pdf. Accessed 8 Mar 2018

[9] Scottish ECT Accreditation Network (SEAN) Annual Report (2015). http://www.sean.org.uk/docs/SEAN-Report-2015-web.pdf. Accessed 8 Mar 2018

[10] Lutchman RD, Stevens T, Bashir A, Orrell M (2001). Mental health professionals’ attitudes towards and knowledge of electroconvulsive therapy. J Ment Health.

[11] Glaser BG, Strauss AL (1967). The discovery of grounded theory.

[12] Charmaz K, Denzin NK, Lincoln YS (2011). Grounded theory methods in social justice research. The 4th Sage handbook of qualitative research.

[13] Strauss A, Corbin J (1998). Basics of qualitative research: techniques and procedures for developing grounded theory.

[14] Charmaz K (2006). Constructing grounded theory. A practical guide through qualitative research.

[15] Bryant A (2003) A constructive/ist response to Glaser. Forum Qualitative Sozialforschung/Forum: Qualitative Social Research, 4(1). http://www.qualitative-research.net/index.php/fqs/article/view/757/1643. Accessed 19 Jan 2018

[16] Dey I (1999). Grounding grounded theory guidelines for qualitative inquiry.

[17] General Medical Council (2013) Good Medical Practice. http://www.gmc-uk.org/guidance/good_medical_practice.asp. Accessed 8 Mar 2018

[18] National Institute of Clinical Excellence (NICE) (2018) Shared decision-making. https://www.nice.org.uk/about/what-we-do/our-programmes/nice-guidance/nice-guidelines/shared-decision-making. Accessed 17 Jan 2018

[19] Mohtashemi R, Stevens J, Jackson P, Weatherhead S (2016). A qualitative exploration of psychiatrists’ understanding and use of psychological formulation. BJPsych Bulletin.

[20] National Institute for Health and Clinical Excellence (NICE) (2011). Service user experience in adult mental health: improving the experience of care for people using adult NHS mental health services. CG136.

[21] Loos S, Clarke E, Jordan H, Puschner B, Fiorillo A, Luciano M, Magyar TIE, Krogsgaard-Bording M, Østermark-Sørensen H, Rössler W, Kawohl W, Mayer B, Slade M, CEDAR Study Group (2017). Recovery and decision-making involvement in people with severe mental illness from six countries: a prospective observational study. BMC.

[22] Slade M (2017). Implementing shared decision-making in routine mental health care. World Psychiatry.

[23] United Nations; Human Rights Council (2017) Report of the Special Rapporteur on the right of everyone to the enjoyment of the highest attainable standard of physical and mental health. https://reliefweb.int/sites/reliefweb.int/files/resources/G1707604.pdf. Accessed 31 Jan 2018

[24] Pilgrim D (2008). ‘Recovery’ and current mental health policy. Chron Illn.

